# Changes in Dickkopf-1 (*DKK1*) and Sclerostin following a Loading Dose of Vitamin D_**2**_ (300,000 IU)

**DOI:** 10.1155/2014/682763

**Published:** 2014-11-24

**Authors:** A. Sankaralingam, R. Roplekar, C. Turner, R. N. Dalton, G. Hampson

**Affiliations:** ^1^Department of Clinical Chemistry, St Thomas' Hospital, North Wing, Lambeth Palace Road, London SE1 7EH, UK; ^2^Wellchild Laboratory, The Evelina Children's Hospital, London SE1 7EH, UK; ^3^Metabolic Bone Clinic, Department of Rheumatology, Guy's Hospital, London SE1 9RT, UK

## Abstract

*Background*. Vitamin D is important for bone health, although high loading doses have been associated with an increase in fracture risk. The mechanisms remain uncertain. *Aim*. We hypothesize that supraphysiological concentrations of 1,25 (OH)_2_ vitamin D may inhibit formation by increasing the production of Wnt inhibitors: sclerostin and *DKK1*. *Subjects and Methods*. We measured serum sclerostin and *DKK1* in 34 patients (21 F, 13 M) aged mean (SD) 61.3 (15.6) years with vitamin D deficiency/insufficiency treated with a loading dose of vitamin D_2_ (300,000 IU) intramuscularly. Blood samples were taken at baseline and serially up to 3 months. *Results*. Serum 1,25 (OH)_2_ vitamin D increased markedly at 3 months (mean (SD) baseline 116 (63), 3 months : 229 (142) pmol/L, *P* < 0.001). There was a significant correlation between sclerostin and *DKK1* at baseline (*r* = 0.504, *P* = 0.002) and at 3 months (*r* = 0.42, *P* = 0.013). A significant inverse correlation was observed between sclerostin and eGFR at 3 months (*r* = −0.494, *P* = 0.007). Sclerostin increased significantly at 3 months (*P* = 0.033). In a multilinear regression analysis with % change in sclerostin and *DKK1* as dependent variable, a positive significant association was observed with % change in 1,25 (OH)_2_ vitamin D (*P* = 0.038), independent of changes in PTH and following correction for confounders such as age, gender, BMI, BMD and eGFR. *Conclusions*. Supraphysiological concentration in 1,25 (OH)_2_ vitamin D achieved following a loading dose of vitamin D increases sclerostin and may inhibit Wnt signalling. This may have detrimental effects on bone.

## 1. Introduction

Vitamin D is important for the maintenance of skeletal health. The best-known physiological role of active vitamin D or 1,25 (OH)_2_ vitamin D concerns the maintenance of calcium and phosphate homeostasis by promoting their intestinal absorption, thus ensuring their availability for the skeletal mineralisation process [1]. To achieve this well-established endocrine effect, 1,25 (OH)_2_ vitamin D works in concert with 2 other hormones: parathyroid hormone (PTH) and fibroblast growth factor-23 (FGF-23) [2]. Aside from its well-characterised endocrine pathway, there is now a growing body of evidence which demonstrates that 1,25 (OH)_2_ vitamin D can directly affect bone cell differentiation and function by targeting key genes involved in bone formation and resorption [3]. The targets for 1,25 (OH)_2_ vitamin D include LRP5, the Wnt coreceptor that plays a key role in osteoblast proliferation, differentiation, and function. In addition, other genes which promote osteoclastogenesis are also regulated by 1,25 (OH)_2_ vitamin D and this includes RANKL, produced by osteoblasts which stimulates bone resorption [4].

Moderate vitamin D deficiency is associated with decreased bone mineral density (BMD) and increased risk of fracture, particularly in the elderly [5]. Randomised controlled trials of vitamin D supplementation alone or combined with calcium have been shown to reduce fracture risk, in elderly institutionalized subjects [6]. Taken together these data indicates that vitamin D supplementation in this clinical setting improves BMD and decreases the risk of osteoporotic fracture. It has been postulated that higher serum concentrations of 25 (OH) vitamin D should be achieved to reduce fracture risk, particularly when vitamin D is given alone without calcium, although there is debate over the optimum serum concentration of 25 (OH) vitamin D needed for maximal antifracture efficacy [6]. In addition, specific vitamin D dosing regimen may also be important as studies of vitamin D loading regimes have shown an increased risk in fracture risk, although the biological mechanisms remain unclear [7, 8].

It is plausible that several biological pathways may be implicated as 1,25 (OH)_2_ vitamin D via its receptor; the vitamin D receptor (VDR) can regulate several osteoblastic and osteocytic genes which affect skeletal remodelling [3]. Excessive signalling by supraphysiological concentrations achieved by high loading doses of vitamin D may favour the expression of genes involved in bone resorption which are negative regulators of bone formation. In support of these hypotheses, recent studies have shown that large doses of vitamin D are associated with acute increases in bone resorption, which may be related to vitamin D-induced upregulation of RANKL or proresorptive cytokines [9, 10]. We have shown that bolus intramuscular injections of 300,000 IU of vitamin D_2_ lead to increases in FGF-23 which may impact negatively on bone mineralisation [11]. Another hypothesis is that changes in factors involved in the regulation of the Wnt signalling pathway may also be modulated by excess 1,25 (OH)_2_ vitamin D signalling in osteocytes or osteoblasts.

Proteins such as sclerostin and Dickkopf-1 (*DKK1*) are 2 secreted Wnt signalling antagonists, produced by osteocytes mainly [12, 13]. In animal models, acute as well as chronic PTH administration reduces the expression of sclerostin by osteocytes [14]. In clinical studies, circulating sclerostin has been shown to be inversely correlated with PTH levels and free oestrogen index [15]. Patients with primary hyperparathyroidism have reduced sclerostin and higher* DKK1* concentrations [16]. A recent study showed an increase in serum sclerostin in men only following vitamin D (700 IU/day) and calcium supplementation (500 mg/day) [17].* DKK1* expression in colon epithelial cells has been shown to be upregulated by 1,25 (OH)_2_ vitamin D [18]. In osteoblasts,* DKK1* production is enhanced by glucocorticoids [19]. We can therefore speculate that vitamin D signalling may affect the production of the 2 Wnt inhibitors.

It is biologically plausible that at physiological concentrations, 1,25 (OH)_2_ vitamin D has an anabolic effect on bone metabolism but at supraphysiological concentrations, such as those achieved with very high loading regimes, it may stimulate factors which have a suppressive effect on bone formation. The aim of this study was to determine changes in circulating concentrations of sclerostin and* DKK1* following a loading dose of vitamin D_2_ (ergocalciferol) in subjects with vitamin D insufficiency.

## 2. Material and Methods

### 2.1. Study Design and Subjects

We studied 34 patients (13 M, 21 F) aged mean (SD) 61.3 (15.6) years with vitamin D insufficiency (25 (OH) vitamin D < 50 nmol/L) as determined by the routine automated immunoassay. The current study is a followup of previous work investigating the effects of a loading dose of vitamin D_2_ on circulating concentrations of 1,25 (OH)_2_ vitamin D and FGF-23 in patients with osteoporosis and vitamin D insufficiency in a subgroup of 34 subjects [11]. They were recruited during their follow-up visit at the metabolic bone clinic over 12 months from October 2010 to September 2011 and had complete datasets which included measurement of serum sclerostin and* DKK1*. The serum 25 (OH) vitamin D concentration on routine analysis was mean (SD) 33.8 (15.5) nmol/L. A loading dose of 300,000 IU of Ergocalciferol (vitamin D_2_) intramuscularly was given and all patients were asked to continue with their usual daily maintenance supplements of calcium (1.2 g) and vitamin D_3_ (800 IU). Twenty-five subjects (74%) were on bisphosphonates. The study was approved by the Local Research Ethics Committee of Guy's and St Thomas' Hospital NHS Trust. Written informed consent was obtained from all subjects. Non-fasting blood samples were collected at baseline and the subjects were followed longitudinally when additional samples were obtained at 1, 2, and 3 months. Routine biochemical parameters were analysed immediately while additional serum and plasma samples were frozen and stored at −70°C for subsequent analyses of 25 (OH) vitamin D and 1,25 (OH)_2_ vitamin D by liquid chromatography-tandem mass spectroscopy (LC-MS/MS), sclerostin, and* DKK1*, respectively. A summary of subjects demographics is shown in Table 1.

### 2.2. Routine Laboratory Measurements

Routine biochemical tests including albumin corrected calcium, phosphate, PTH, and creatinine were determined by standard laboratory methods on the Roche Modular Analysers (Roche Diagnostics Limited, West Sussex RH15 9RY, UK). Estimated GFR (e GFR) was derived using the MDRD formula. The biochemical markers of bone turnover, CTX (*β*-Crosslaps) and total procollagen type 1 amino-terminal propeptide (PINP), were measured on the Roche Elecsys 2010 analyser as previously described [11]. The assay CV for plasma *β*-CTX ranged between 2.6% and 3%. The assay CV for P1NP was 5.7% and 4.8% at concentrations of 30.6 and 185 *μ*g/L, respectively. The routine assay for 25-hydroxyvitamin D (25 (OH) vitamin D) was an automated chemiluminescence immunoassay (CLIA) (LIAISON Diasorin Inc., Stillwater, MN, USA). The interassay CV ranged between 9.6% and 11.7%.

25 (OH) vitamin D_2_, 25 (OH) vitamin D_3_, 1,25 (OH)_2_ vitamin D_2_, and 1,25 (OH)_2_ vitamin D_3_ were also analysed at baseline and at 3 months simultaneously by liquid chromatography-tandem mass spectroscopy (LC-MS/MS), the gold standard methodology, as previously described [11]. This method, unlike the immunoassays, is not affected by vitamin D binding protein concentrations and is able to equally detect 25 (OH) vitamin D_2_ and 25 (OH) vitamin D_3_. The recommended National Institute of Standards and Technology (NIST) standard reference material (SRM) 972 was used as calibrator for the 25 (OH) vitamin D assay, hence avoiding issues surrounding assay standardisation as reported for certain commercial immunoassays. Calibrators for 1,25 (OH)_2_ vitamin D were obtained from Sigma-Aldrich, Dorset, UK. Interassay CVs ranged between 5.6% and 6.6% for 1,25 (OH)_2_ vitamin D_3_ and 6.2–13.5% for (OH) vitamin D_2_ and 25 (OH) vitamin D_3_ concentrations.

*DKK1* was measured by an ELISA (DuoSet ELISA, R&D Systems Europe, Ltd., Abingdon OX14 3NB, UK) according to the manufacturer's instructions. The 96-well microtitre plates were coated with 100 *μ*L of anti-*DKK1* monoclonal antibody diluted to 8.0 *μ*g/mL. The detection antibody (goat anti-human* DKK1*) was diluted to a concentration of 100 *μ*g/mL. The minimum detection limit was 631 pg/mL and the assay CV was 1.45% and 1.2% at* DKK1* concentration of 889 pg/mL and 3254 pg/mL, respectively, the same batch to minimise variability. Sclerostin was measured by an immunocapture enzyme assay (TECO medical Group, Quidel Corporation, San Diego, USA). The minimum detection limit of the assay is 0.008 ng/mL. Assay CV was 6.2% at sclerostin concentration of 0.24 ng/mL.

### 2.3. Dual Energy X-Ray Absorptiometry (DXA)

Bone mineral density was measured at the lumbar spine (LS) and total hip (TH) at baseline by DXA using the Hologic Discovery scanner (Hologic Inc., Bedford, MA). The CV for BMD measurement was 1.6% at the LS and TH and 2.5% at the FN.

### 2.4. Statistical Analyses

Mean and standard deviation (SD) were derived for all continuous variables. Nonparametric data were log-transformed to normalize the data. Univariate analysis, using Pearson's correlation or Spearman's rank correlation, was used to explore the relationship between* DKK1* and sclerostin, with eGFR, PTH, and vitamin D metabolites at baseline and at 3 months. Differences between the biochemical parameters at baseline and 3 months were determined using the student paired *t* test. Percentage change in* DKK1* at 1, 2, and 3 months compared to baseline was analysed using ANOVA. Multilinear regression analysis was used to explore the association between changes in sclerostin and* DKK1* and changes in 1,25 (OH)_2_ vitamin D after adjustment for age, gender, BMI, and BMD at the LS and TH and PTH. All statistical analyses were performed using IBM SPSS Statistics 20 (Mac). A *P* value of <0.05 (95% confidence interval) was considered as statistically significant.

## 3. Results

### 3.1. Changes in Biochemical Parameters following Vitamin D_2_

There was a marked increase in 25 (OH) vitamin D and 1,25 (OH)_2_ vitamin D, measured by LC-MS/MS, at 3 months as shown in Table 2. No significant differences were observed between PTH, serum calcium, and the bone turnover markers at 3 months compared to baseline in this subgroup. None of the study participants became hypercalcemic. Serum phosphate increased significantly (*P* = 0.039) (Table 2). There were no significant differences in sclerostin at baseline and at 3 months between men and women.

### 3.2. Wnt Inhibitors: Sclerostin,* DKK1*

**Figure 1:**
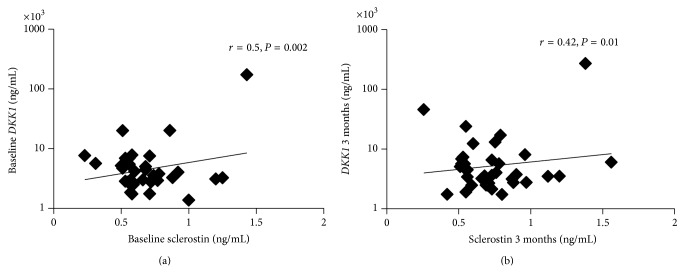
The correlation between circulating sclerostin and* DKK1* at baseline (a) and at 3 months (b) following a loading dose of vitamin D_2_ (300,000 IU).

There was a small increase in* DKK1* concentrations between baseline and at 3 months, following the bolus dose of vitamin D_2_, although this failed to reach significance (*P* = 0.2) Table 2. In contrast, sclerostin increased significantly at 3 months (*P* = 0.033) Table 2. Sclerostin also increased in the subgroup of patients who were not on treatment with bisphosphonates (*n* = 9), although the results failed to reach significance (baseline: 0.553 (0.13), 3 months: 0.628 (0.16) ng/mL, *P* = 0.16). Univariate analyses showed a significant positive correlation between sclerostin and* DKK1* at baseline (*r* = 0.504, *P* = 0.002) as illustrated in Figure 1(a) and 3 months (*r* = 0.42, *P* = 0.013) Figure 1(b). There was no significant relationship between PTH, 1,25 (OH)_2_ vitamin D, and 25 (OH) vitamin D with sclerostin or* DKK1* at baseline and 3 months. A significant inverse correlation was observed between sclerostin and eGFR at 3 months (*r* = −0.494, *P* = 0.007). Only the association between sclerostin and* DKK1* remained significant at baseline (*P* < 0.001) and 3 months (*P* = 0.003) following multilinear regression analyses and adjustment for age, gender, PTH, and vitamin D metabolites.

### 3.3. Changes in Sclerostin and* DKK1* following a Loading Dose of Vitamin D_2_

In univariate analysis, a positive correlation was observed between % change in sclerostin and 1,25 (OH)_2_ vitamin D from baseline. This tended towards significance (*r* = 0.324, *P* = 0.07). The mean (SEM) of the combined % change in the 2 Wnt inhibitors were 23% (7.2), although the % change in* DKK1* at 3 months was lower than sclerostin (*DKK1*: 10% (5.3), sclerostin 13% (5)). We observed a trend towards a small and gradual increase in* DKK1* (1 month: 2% (3.9), 2 months: 6% (4.1), and 3 months: 10% (5.3), *P* = 0.2). In a multilinear regression analysis with % change in sclerostin and* DKK1* as dependent variable, following correction for confounders such as age, gender, BMI, BMD, and GFR, we found a positive significant association with % change in 1,25 (OH)_2_ vitamin D independent of changes in PTH (*P* = 0.038) Table 3.

## 4. Discussion

We have shown that increases in 1,25 (OH)_2_ vitamin D following a loading dose of vitamin D_2_ are associated with a significant increase in serum sclerostin. In multilinear regression % changes in the Wnt inhibitors, sclerostin and* DKK1* was significantly associated with increases in 1,25 (OH)_2_ vitamin D, independent of PTH.

Vitamin D supplementation reduces the risk of fractures in populations with a high prevalence of vitamin D deficiency or insufficiency [6]. There are however several studies, particularly when loading doses of vitamin D were given, where “U” or “J” shape association between bone health and vitamin D has been reported [7, 8]. Thus, at lower doses, vitamin D has a protective or anabolic effect on bone health, but it could exert adverse or catabolic effects at higher doses. The mechanisms for such an effect are still not completely understood, although studies have shown an increase in bone resorption, following high bolus doses (300,000–600,000 IU) [9, 10]. This has been postulated to be due to an increase in RANKL production, although not demonstrated due to limitations in the measurement of circulating RANKL concentrations. We have previously demonstrated increases in 2 potent proresorptive cytokines; tumour-necrosis factor-*α* (TNF-*α*), and interleukin-1*β* (IL-1*β*) following a loading dose of 300,000 IU of vitamin D_2_ [20]. It is also possible that supraphysiological concentrations, such as those achieved in our study may affect bone formation, for instance increases in FGF-23, as previously documented [11], could result in reductions in bone formation and impairment in mineralisation. The marked increases in serum 25 (OH) vitamin D are in contrast to findings from a previous study. This is likely due to assay differences as we used LC-MS technology which detects 25 (OH) vitamin D_2_ and 25 (OH) vitamin D_3_ equally in contrast to immunoassays which underestimate 25 (OH) vitamin D_2_ [21].

1,25 (OH)_2_D has been shown to regulate low density lipoprotein receptor-related protein-5 (LRP5), the Wnt coreceptor essential for Wnt signalling and bone formation [3]. We hypothesised that high concentrations of 1,25 (OH)_2_ vitamin D could inhibit this pathway by up-regulating the Wnt inhibitors; sclerostin and* DKK1* which bind to LRP5/6. Both sclerostin and* DKK1* are inhibitors of bone formation, although their tissue distribution is different. Sclerostin is primarily expressed by osteocytes or late osteoblasts whereas* DKK1* has a wider tissue distribution [12, 13]. In adults, the expression of* DKK1* is abundant in osteoblasts and maturing osteocytes, although it is also expressed by other cell types including platelets [22]. In disease,* DKK1* is expressed by synovial fibroblasts, plasma cells, and colon cancer cells [18, 23, 24]. In our study, we observed a close correlation between both Wnt inhibitors at baseline and after high dose vitamin D. This finding has not been demonstrated in all studies [25]. One explanation for the divergence is the difference in assays used as they may measure different parts or fragments of the sclerostin and* DKK1* molecule.

Sclerostin and* DKK1* have been shown to be differentially regulated by treatment agents used in osteoporosis with increases in sclerostin and either no change or decreases in* DKK1* seen with antiresorptive agents such as bisphosphonates and denosumab, respectively [26–28]. On the other hand, with anabolic agents, such as teriparatide, decreases in sclerostin with later increases in* DKK1* following prolonged treatment has been observed [26]. Similar findings have also been seen in chronic elevations in PTH as in primary hyperparathyroidism [16]. Glucocorticoid treatment also has a differential effect on sclerostin and* DKK1* [29]. Data from these studies suggest that early changes in sclerostin may be followed by a later reciprocal change in* DKK1* which acts as a counter-regulatory mechanism. The findings also suggest that* DKK1* response appears to be slower with significant changes occurring at 12 months and 18 months following teriparatide and denosumab, respectively. We observed a significant increase at 3 months in sclerostin following high dose vitamin D_2_. Changes of similar magnitude as in our study have been reported following calcium and vitamin D supplementation for 2 years in men only, although the dose of vitamin D was lower than in our study and 1,25 (OH)_2_ vitamin D was not measured [17]. We did not observe any difference in sclerostin at baseline and 3 months between men and women. More profound increases in sclerostin have been observed at earlier time points following potent antiresorptive agents such as intravenous zoledronate [30] or denosumab [27]. In the case of denosumab, an average increase of sclerostin of 29% was observed at 6 months which was maintained over 3 years. In contrast, with zoledronate, the increase in sclerostin was seen at early time points (7 days) with decline at 1 month and returning to baseline at 12 months [28]. In our study, we do not have sclerostin measurements at serial time points and it is plausible that increases may have been larger at earlier time points. There was a trend towards a gradual increase in* DKK1* which may be slower to respond compared to sclerostin. Previous studies in a colon cell line showed* DKK1* expression to be induced following treatment with 1,25 (OH)_2_ vitamin D at high concentrations (10^−7 ^M) [18]. Both Wnt inhibitors appear to be upregulated by supraphysiological concentrations of 1,25 (OH)_2_D implying that inhibition of the Wnt signalling pathway may be implicated in the catabolic actions of high levels of 1,25 (OH)_2_D. We did not see any significant change in P1NP as the majority of the patients were already established on bisphosphonate at the time of administration of the loading dose of vitamin D_2_ and this may have masked the suppressive effect of sclerostin on bone formation.

Changes in the combined Wnt inhibitors were significantly associated with the increases in 1,25 (OH)_2_ vitamin D. This appears to be independent of PTH and GFR, suggesting an independent effect of 1,25 (OH)_2_ vitamin D on sclerostin in particular. We also adjusted for bone density as studies suggest that circulating sclerostin may be related to the osteocyte pool [31]. A recent animal study in knockout sclerostin mice shows that sclerostin may influence mineral metabolism and may have a direct inhibitory effect on 25-hydroxyvitamin D 1*α*-hydroxylase gene (cyp*27B1*) mRNA expression [32]. Furthermore, the study also shows that the absence of sclerostin leads to reduced FGF-23 concentrations suggesting that sclerostin may regulate FGF-23 [29]. We have previously demonstrated significant rises in serum phosphate and FGF-23 following high dose vitamin D_2_ [11] Taken together, these data would implicate sclerostin as another player in the feedback loop involving phosphate, FGF-23, and 1,25 (OH)_2_ vitamin D. Further studies are required to explore this interesting possibility.

There are several limitations to our study. Firstly, we did not measure sclerostin at earlier time points. Secondly, as* DKK1* appears to take longer time to respond to changes in 1,25 (OH)_2_ vitamin D, measurement at later time points would have been useful to compare differences in time course between sclerostin and* DKK1*. We could not determine whether the changes in the Wnt inhibitors were associated with changes in bone formation or bone turnover as most of the patients were on bisphosphonates. Bisphosphonates have also been shown to increase sclerostin, although the majority of patients were already established on bisphosphonates at study entry and none of the patients started bisphosphonates during the 3 months study period. Nevertheless, our data show that the supraphysiological concentrations in 1,25 (OH)_2_ vitamin D achieved following a loading dose of vitamin D may inhibit Wnt signalling and this may have adverse effects on the skeleton. Further studies are needed to assess the effects of routine administration of bolus doses of vitamin D on bone health.

## Figures and Tables

**Table 1:** Summary of subjects demographics. Data are shown as mean (SD).

Number of study participants	34
(M/F)	(13/21)
Age (years)	61.4 [15.3]
BMI (kg/m^2^)	26.6 [4.8]
BMD lumbar spine (g/cm^2^)	0.78 [0.11]
*T*-score lumbar spine	−2.54 [0.96]
BMD hip (g/cm^2^)	0.77 [0.116]
*T*-score hip	−1.48 [0.81]
Previous fractures (%)	21 (62%)
Treatment with oral bisphosphonate (%)	25 (74%)

**Table 2:** Biochemical parameters and circulating concentration of sclerostin and *DKK1*.

Biochemical analytes (mean [SD])	Baseline	1 month	2 months	3 months
Calcium (mmol/L)	2.29 [0.13]	2.32 [0.12]	2.33 [0.10]	2.35 [0.13]
Phosphate (mmol/L)	1.03 [0.24]	1.11 [0.22]^*^	1.14 [0.22]^*^	1.11 [0.19]^*^
eGFR (mL/min)	97 [36]	88 [31]	93 [34]	86 [25]
PTH (ng/L)	56 [58]	46 [30]	45 [29]	46 [28]
25 (OH) vitamin D (nmol/L) (immunoassay)	33.6 [14.7]	46.1 [20.3]^**^	51 [22]^**^	54 [20]^**^
Total 25 (OH) vitamin D (nmol/L) (LC-MS/MS)	48.2 [18]	—	—	78.7 [25.4]^**^
Total 1,25 (OH)_2_ vitamin D (pmol/L) (LC-MS/MS)	116 [63]	—	—	229 [142]^**^
Plasma *β*CTX (*µ*g/L)	0.18 [0.18]	0.19 [0.21]	0.21 [0.22]	0.19 [0.13]
Serum P1NP (*μ*g/L)	34 [33]	33 [24]	31 [19]	30.5 [18]
Wnt inhibitors (mean (SEM))				
Sclerostin (ng/mL)	0.694 [0.04]	—	—	0.753 [0.04]^*^
*DKK1* (ng/mL)	9908 [5015]	9572 [4978]	12875 [7319]	13047 [7855]

^*^ *P* < 0.05, ^**^ *P* < 0.01 v/s baseline.

**Table 3:** Multilinear regression analysis of the % change in the Wnt inhibitors, sclerostin and *DKK1* as dependent variable and % change in 1,25 (OH)_2_ vitamin D as independent variable, following correction for age, gender, and BMD. Highlighted bold is the significant variables. Dependent variable: combined % change in *DKK1* and sclerostin.

Variables	*β*-coefficients	*T* value	*P* value
Age	0.404	1.524	0.151
Gender	0.161	0.590	0.565
BMI	0.155	0.535	0.602
BMD lumbar spine	0.175	0.622	0.545
BMD total hip	−0.127	−0.301	0.768
Baseline GFR	0.154	0.524	0.609
**% change in 1,25** (**OH**)_2_ ** vitamin D**	**0.611**	**2.306**	**0.038**
% change in PTH	−0.293	−1.054	0.311
% change in 25 (OH) vitamin D	−0.491	−1.654	0.122
